# Understanding the Phenomenon of Binge-Watching—A Systematic Review

**DOI:** 10.3390/ijerph17124469

**Published:** 2020-06-22

**Authors:** Jolanta A. Starosta, Bernadetta Izydorczyk

**Affiliations:** Institute of Applied Psychology, Faculty of Management and Social Communication, Jagiellonian University, 30-348 Krakow, Poland

**Keywords:** binge-watching, behavioural addiction, new technologies, motivation, personality traits

## Abstract

Binge-watching is a relatively new behaviour pattern whose popularity has been increasing since 2013, ultimately to become one of the most popular ways of spending free time, especially among young people. However, there is still a dearth of research on this phenomenon. The aim of this study is to present the current understanding and psychological conditions of binge-watching, as provided in the research papers published between 2013 and 2020. This systematic review, including 28 articles, addresses different approaches to defining this behaviour, diverse motivations, personality traits, and risks of excessive binge-watching. Its results imply that there are two perspectives in understanding binge-watching. The first is related to entertainment, positive emotions, cognition, and spending free time. However, the second perspective emphasises the negative outcomes of excessive binge-watching and symptoms of behavioural addiction. There is undoubtedly a need for further research to be conducted on diversified populations to reach more profound understanding of binge-watching behaviour patterns.

## 1. Introduction

Binge-watching is a relatively new behavioural phenomenon, which is defined as watching between two and six episodes of a TV show in one sitting [1]. This behaviour started to gain popularity as a result of the development of multiple on-demand streaming platforms such as Netflix, Hulu, HBO GO, Amazon Prime, Disney+, Crunchyroll, and Apple TV [2]. In 2013, Netflix created new ways of consuming TV shows, where the viewer can choose from extensive and diverse content and watch as many episodes of TV series as they want. Furthermore, the whole season of a TV show is available at once. The viewer does not have to wait a week for the release of the next episode, which is characteristic of traditional television [3]. However, one should also mention that binge-watching existed earlier and manifested itself as watching multiple episodes in TV marathons, on VHS, DVD, DVR—digital video recorder, or VOD—video on demand [4,5,6,7]. The popularity of binge-watching increased between 2011 and 2015, ultimately to become a normal way of consuming TV series among general audiences [8]. The latest data released by Netflix in the third quarter of 2019 show that this streaming platform has over 167 million paying subscribers [9]. This number increased rapidly from 5 million in 2012 to more than 167 million in 2020 [9,10]. It is worth mentioning that Netflix is available in 190 countries. The research conducted in 2013 shows that 62% of the American population admit that they binge-watch regularly [11]. However, according to the data from YouGov Omnibus, 58% of Americans claim that they have binge-watched [12]. Furthermore, binge-watching is a regular way of consuming TV shows for 72% of those surveyed. Multiple studies imply that people at the age of 18 to 39 are more likely to binge-watch than older people [10,13]. In 2015, Moore [14] found that binge-watching is a gender-neutral phenomenon. The differences between men and women manifest themselves in their TV show preferences. Women tend to choose comedies and dramas more, while men decide to watch fantasy or sci-fi series more frequently. The latest research shows that women are more likely to watch cable TV than men [10]. Men might be more inclined to stream online content. However, the Netflix user base comprises 49% men and in 51% women. Moreover, people can binge-watch at any place, for example as they commute to work, using diverse electronic devices such as mobile phones, laptops, or tablets [15]. Furthermore, another survey indicates that people tend to binge-watch alone, and that more than 70% of them lose control in terms of how many episodes they have actually watched in one sitting [13]. It is also important to mention that, since most people binge-watch in solitude, this can be conditioned by specific personality traits, while excessive or problematic binge-watching may lead to further isolation and a feeling of loneliness [16,17].

Binge-watching has undoubtedly become the common and entertaining way of consuming media content, such as TV series, for the contemporary audience. The phenomenon has been observed since 2013, but the research concerning its psychological conditions, such as personality traits as well as motivational and emotional mechanisms, is still scarce. Recent literature emphasises that excessive forms of binge-watching could be similar to such behavioural addictions as video games/internet addiction or problematic social media use [8,18,19,20]. This highly immersive behaviour provides immediate gratification, and thus it may lead to the loss of self-control and spending much more time on watching TV series than the person originally wanted [19,21,22]. Research also show the relation between this type of sedentary activity and negligence of work or social relationships, lack of sleep, bedtime procrastination, overweight, or increase in unhealthy food consumption [23,24,25,26,27].

The aim of this systematic review is to present all the recent research on the phenomenon of binge-watching and its psychological conditions in order to provide better comprehension of this common phenomenon. Further subjects the study addresses are the addictive nature of this behaviour and the above-mentioned risks for the viewers, which could be important for the prevention of mental disorders as well as for better understanding of excessive forms of binge-watching.

## 2. Materials and Methods

### 2.1. Search Strategy

This systematic review was conducted according to the Preferred Reporting Items for Systematic Reviews and Meta-Analyses—PRISMA. The aim of the screening was to capture all the relevant studies concerning the phenomenon of binge-watching. The search strategy combined key terms such as: “binge-watching” AND “addiction”. However, due to the small number of articles (133, and 14 after duplicates and unscientific articles have been removed) found by this algorithm, and in order to avoid the risk of bias in the presentation of the results of the systematic research on the binge-watching phenomenon, a decision to change the algorithm was made. The final algorithm adapted in the database search stage was: “binge-watching” OR “binge-viewing”. These terms were entered in the search using EBSCO (Web of Science, Scopus, PsychArticles, PsychInfo) and Google Scholar. Consequently, 2007 citations from EBSCO, 360 citations from Google Scholar, and 6 articles from secondary search were retrieved using this procedure.

### 2.2. Inclusion Criteria

The following criteria of inclusion were adapted:

(1) The articles were published between 2013 and March 2020. As already mentioned in the introduction, the binge-watching phenomenon dates back to 2013, and it has been growing in popularity ever since, ultimately to become one of the most common ways of free-time spending. However, it should be mentioned that other forms of this phenomenon could be studied even before 2013 under the research on television addiction.
(2) The articles concerned binge-watching and its psychological conditions (for example motivation, engagement, personality traits, and relations with mental health disorders).
(3) The articles were published in English.
(4) The relevant studies were of empirical and quantitative nature.

### 2.3. Search and Screening

**Figure 1:**
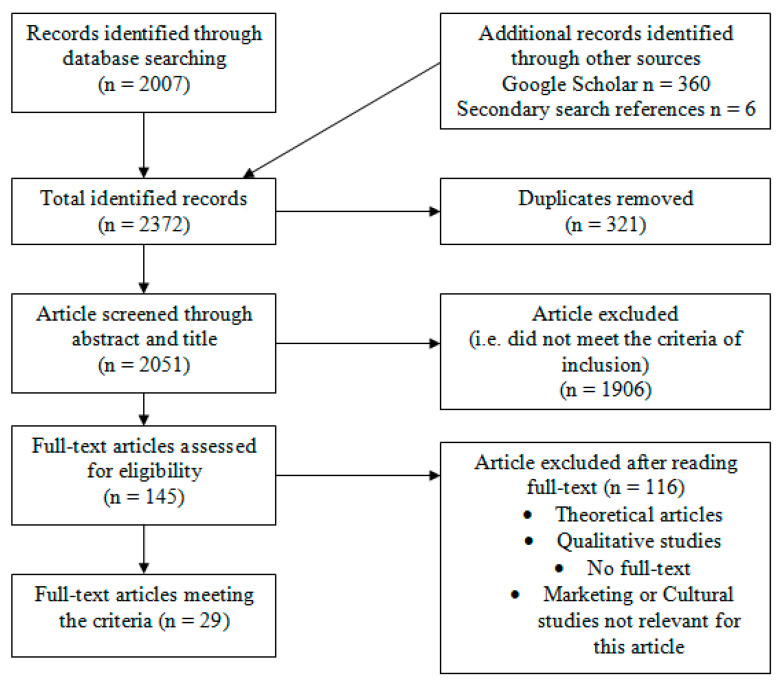
Flowchart of the study screening and selection process.

A total of 2373 sources were identified through electronic database searching. Once 321 duplicates and 1906 articles that did not conform to the inclusion criteria were removed, 145 full-text articles were assessed for eligibility. However, 116 articles were excluded after the reading because some of them emphasised the marketing or cultural (art and film studies related) aspect of binge-watching, which was not relevant to this systematic review, while others addressed theoretical or qualitative studies. In the end, 29 full-text articles met all the review criteria. Figure 1 provides a flowchart outlining the search for the studies.

## 3. Results

### 3.1. Characteristics of the Studies on the Binge-Watching Phenomenon Included in the Systematic Review

The studies included in this systematic review were developed between 2014 and 2019, which is understandable on account of the increasing popularity of binge-watching, after this behaviour was redefined in 2013 when Netflix created a new way of consuming media such as TV series. Most of the studies were conducted in the USA (*n* = 16, 55%), which is natural given the fact that streaming platforms have always been most popular and most accessible in America. Netflix has been present in the USA since 2013, while in many European countries, for example, it became available in 2016. Considering the foregoing facts, it is not surprising that the majority of the studies in question were conducted in the United States of America. The research on the subject of binge-watching was also performed in European countries (32%) such as Belgium (*n* = 3), the Netherlands (*n* = 2), Poland (*n* = 1), Portugal (*n* = 1), the United Kingdom (*n* = 1), Germany (*n* = 1), and Hungary (*n* = 1). Some studies were also conducted in South Korea (*n* = 1), Tunisia (*n* = 1), and United Arab Emirates (*n* = 1). A total of 32,464 participants took part in the research covered by this systematic review. Women comprised the majority of the research subjects in most of the studies (*n* = 24). Furthermore, the average age of the participants ranged between 18.54 and 47. However, most of the persons who participated in the majority of the studies were aged between 18.54 and 29 [16,18,19,20,24,25,28,29,30,31,32,33,34,35,36,37,38,39,40,41] Only in six studies, the average age of those surveyed was higher, namely between 30 and 47 years [4,22,42,43,44,45]. Some articles addressed marital status in their sociodemographic variables, and the results show that most of those surveyed were unmarried and had no children [22,28,31,34,35,42,46]. Moreover, the majority of the research subjects in all of the studies covered by this systematic review were students. Another sociodemographic variable included in some studies was the household income [31,34,40,46] Only Walter et al. [35] decided to address the aspect of religion in her research, and its results show that most of those surveyed were Christians. The subject of the research typically included such psychological conditions of binge-watching such as motivation, personality traits, transportation into the narrative, emotional regulation, parasocial relationships, and engagement. The research also focused on the negative outcomes of the problematic binge-watching behaviour patterns such as lack of control, unhealthy diet, sleeping problems, guilt, relation with affective disorders, and neglect of duties. Furthermore, it also focused on the relation between binge-watching and depression, loneliness, anxiety, self-control, and impulsiveness. The above-mentioned characteristics of the research covered by the systematic review have been presented in Table 1.

### 3.2. Methodological Evaluation of the Research Covered by the Systematic Review

All studies covered by the systematic review had clear aims. The methods used in the research were thoroughly described and were characterised by satisfactory psychometric properties such as reliability and validity [4,16,18,19,20,22,24,25,29,30,31,32,33,34,35,36,37,38,40,43,45,46,48]. Four studies included descriptions of the methods used and the survey design but did not provide the reliability coefficient—Cronbach’s α [28,39,41,44]. Most of the studies used non-probability and purposive sampling [4,16,18,19,20,22,24,25,28,29,30,31,32,33,34,35,36,37,38,39,40,41,43,45,46,47]. Random sampling was only applied in two studies [42,44]. The surveys differed from one another in terms of the sample size. One study covered by the systematic review was based on an experimental approach [48]. Five were conducted on a group larger than one thousand persons [19,20,30,37,47]. In eighteen studies, the sample size ranged between one hundred and one thousand participants [4,16,18,24,25,28,29,31,32,34,35,36,38,40,42,43,44,45,46], while five of them were conducted on a sample composed of less than one hundred individuals [22,33,39,41,48]. All the results addressed further in this paper, in the results section, are statistically significant unless otherwise stated.

### 3.3. Definition of Binge-Watching

When scientists refer to binge-watching, they typically consider the number of episodes watched during a session, the frequency of binge-watching sessions, and the content being watched. Most studies define binge-watching as watching multiple episodes of TV series in one sitting, or use the definition created by Netflix, whereby binge-watching is watching between 1 to 6 episodes in one sitting [2]. Moreover, researchers tried to count the number of episodes watched by viewers, i.e., from 1 to 3 [28,29], from 1 to 6 [30,31,32], 3-plus episodes [19,33,42], or even more [14,20,24,35,47]. Another approach was to measure the exact time spent by viewers on binge-watching [18,31]. Rubenking and Bracken [43] focused on the length of episodes, and defined binge-watching as watching three to four or more thirty-minute-long episodes of TV series or watching three or more one-hour-long episodes. Some studies disregarded the types of binge-watchers with reference to the number of episodes watched [20,31]. The higher the number of episodes a person watched, the more problematic their behaviour was. With regard to frequency, scientists measured the number of binge-watching sessions in which individuals engaged per day, week, and month [30]. On the other hand, some studies indicate that the term binge-watching should be limited to watching episodes of the same TV series [18,19,31], while other researchers claim that viewers can binge-watch multiple episodes of different TV series in a short time [4,47]. However, the diverse definitions of binge-watching usually include expressions such as “in one sitting” or “in one session”, which may indicate that most scientists emphasise that watching multiple episodes of TV series should happen in a row [19,30,31,37,43]. The foregoing highlights some difficulties and the diversification of the approaches to defining the binge-watching phenomenon. The general conclusion that may be drawn with reference to the above-mentioned research is that binge-watching can be defined as watching multiple episodes of a TV show in one sitting.

### 3.4. Motivation

The motivation for watching TV series is the most extensively studied psychological condition of binge-watching. Most of the research refers to the Uses and Gratification Theory, which explains that individuals use media such as the internet, television, and social media to satisfy their needs [43]. There are many reasons why people binge-watch. The basic explanation refers to instant gratification and hedonistic needs related to entertainment, engagement, and relaxation [4,19,20,29,30,31,38,39,42,43]. Individuals may use binge-watching to enhance or maintain positive affect or to obtain stimulation. One can assume that people driven by hedonistic and obsessive motivations would seek instant gratification and would probably binge-watch as soon as this activity becomes available [4,37].

Another motivation for binge-watching is of a social nature. People binge-watch to make social connections, to become part of the group or the fandom, to feel accepted by their peers [20,31]. Moreover, studies conducted by Shim and Kim [34] show that people tend to have more motivation to binge-watch a TV series if it is recommended by others.

It seems important to mention that the results obtained by Conlin, Billings, and Averset [44] emphasise the statistically significant relation between FOMO (fear of missing out) and the binge-watching phenomenon. Results show that increased FOMO was a significant predictor for binge-watching especially dramatic series to “catch up” with the narrative and join the cultural conversation. Scientists emphasise that people binge-watch new TV series as soon as possible because they do not want to be ostracised in future conversations with others. Moreover, the authors indicate that high FOMO makes people binge-watch TV shows to avoid spoilers, which could potentially decrease the enjoyment of a series.

Furthermore, the results obtained by Panda and Pandey [31] imply that social engagement, escape, influence of advertising, and accessibility are the main motives behind the intention to spend more time binge-watching. Surprisingly, the scientists indicate that people who experience negative gratification and feel anxious or nervous after a binge-watching session are more likely to spend more time doing it, thus becoming increasingly addicted to this behaviour. Schweidel and Moe [47] also emphasise the effect of engagement in such behaviour on the amount of time spend on binge-watching. Obtained results imply that people who tend to highly engage in binge-watching display a tendency to watch additional episodes of the same series and are more prone to consuming more episodes over a given period of time. Such individuals tend to spend more time watching TV series and are highly inclined to quickly beginning the next session. However, exposure to advertisements during a binge-watching session discouraged people from viewing further on.

Moreover, there is a significant relation between binge-watching and compensatory motivations, where binge-watching becomes a way to escape reality and avoid problems or negative emotions [19,20,31,39]. Panda and Pandey [31] imply that people tend to binge-watch more to escape reality, which can lead to decrease of other, more adaptive way of coping with negative emotions. Furthermore, study implies there is a significant correlation between binge-watching and motivation to deal with loneliness, thus TV series or fictional characters become viewer’s companions in solitude [20]. It is also important to mention that compensatory motivations are typical of individuals who display problematic binge-watching behaviour patterns [20,30,31]. Moreover, results obtained by Rubenking and Bracken [43] and Flayelle et al. [30] show that binge-watching of TV series can be a strategy used to regulate negative and aversive emotions. Furthermore, Castro et al. [39] also highlights the change in affect after a binge-watching session. It may be assumed that some people binge-watch to regulate their own emotions and to cope with their problems.

The results obtained by Sung et al. [40] show that persons with low binge-watching severity are usually motivated only by the entertaining quality of this behaviour. However, results of regression analysis imply that using binge-watching to pass the time is typical of people who binge-watch at a high frequency. Moreover, the research conducted by Rubenking and Bracken [43] indicates that binge-watching has become an entrenched habit in the group of college students.

On the other hand, Conlin [4] and Walter et al. [35] have presented a different approach to understanding the motivation behind and attractiveness of binge-watching for the common viewer. Binge-watching can be a highly immersive experience of transportation into the fictional world, which is usually related to high emotional and cognitive engagement with the narrative as well as identification with characters. Moreover, Pittman and Steiner [42] have highlighted two types of motivation: narrative transportation and narration completion. Their study shows that individuals with higher motivation to complete the narration are less likely to regret the amount of time spent on binge-watching. The scientists explain that individuals with high motivation to complete narration tend to be more self-aware and more attentive, which results in higher self-control and lower regret. What this study also implies is that the more people binge-watch, the more the narrative transportation becomes their motivation. On the other hand, results of research conducted by Erickson, Dal Cin, and Byl [48] imply that binge-watchers tend to create stronger parasocial relationships with their favourite character than non-binge-watchers. This attachment to the fictional characters was also highlighted in research conducted by Wheeler [16]. These results imply that this kind of relationship may be characteristic for people with anxious attachment. However, this finding should be approached with caution, because it has been obtained as a result of master thesis research [16]. These findings are covered by this systematic review due to the novelty of the research subject and dearth in the literature.

Furthermore, some individuals are characterised by self-development and cognitive motivation to gain information or knowledge by watching TV series [20,34,37].

### 3.5. Personality Traits

Pittman and Steiner [42] claim that people who binge-watch are more neurotic, less agreeable, less conscientious, and less open to new experience. These conclusions have been confirmed in the research by Govaert [28] and Tóth-Király, Tóth-Fáber, Hága, and Gábor [37]. It can be assumed that people who display a tendency to excessive binge-watching can be more prone to feeling negative emotions, such as sadness or anxiety, may have low tolerance for stressful situations, low self-esteem, and inclination to self-criticism. These factors are usually predictors of internet and computer addiction [49]. Research also shows that problematic binge-watchers are characterised by low conscientiousness, which can be related to low academic achievements, procrastination, and duty avoidance, but also that binge-watchers cope with stress using avoidance and emotional coping, instead of task-oriented coping [28,36,41]. Other studies imply that, besides high neuroticism and low conscientiousness, impulsivity and lack of self-control are further statistically significant predictors of problematic binge-watching [18,19,28,30]. Riddle et al. [18] have emphasised that high impulsivity is related to unintentional binge-watching, this being the main condition to increase the risk of behavioural addiction. Flayelle et al. [19,30] stress that urgency is one of the most statistically significant impacts of the binge-watching behaviour. Urgency is related to lack of control and tendency to act rashly. An individual may decide to act impulsively in response to both positive and negative emotions. It can be assumed that binge-watching is used to deal with aversive emotions such as sadness or anxiety as well as to enhance positive emotions and attain instant gratification. In the literature, such a tendency has been marked as a risk factor of maladaptive strategies of emotional regulation characteristic of behavioural addictions [19,21]. Further personality traits that may be significant for enhanced understanding of binge-watching are sensation-seeking [34]. The results obtained by Flayelle et al. [30] show that individuals with a high level of sensation-seeking can be prone to excessive TV viewing because of the constant search for arousing and exciting stimuli. It can be assumed that people who display high impulsivity, urgency, and sensation seeking may be more prone to the addictive nature of TV series binge-watching [18,19,30]. Additionally, the need for cognition, understood as an individual’s inclination to engaging in elaborated thinking, has proved to be significant for the binge-watching tendency [34]. Watching multiple episodes of TV series seems to require a high level of cognitive resources. Shim and Kim [34] imply that individuals with a higher need for cognition tend to engage in the binge-watching behaviour more frequently than people with a low need of cognition. On the other hand, Conlin [4] has emphasised that transportability, i.e., the ability to experience immersion into the narrative, is one of the most significant predictors of binge-watching. Results of this study also suggest that another personality trait characteristic of people who display the tendency toward binge-watching is fantasy empathy, described as the ability to feel the emotions of fictional characters, being one of the predictors of character identification and transportation into the narrative.

### 3.6. Binge-Watcher Profiles

The research conducted by Flayelle et al. [30] implies that one can speak of four profiles of binge-watchers: avid binge-watchers, recreational TV series viewers, unregulated binge-watchers, and regulated binge-watchers. The first group is characterised by the highest level of sensation seeking and motivation for TV series watching compared to other groups, but also by high urgency and emotional reactivity. The lowest motivation for watching TV shows and lesser time spending on this activity are characteristic of the recreational TV series viewers. However, the unregulated binge-watchers attain the highest scores in terms of the motivations (emotional enhancement and coping mechanism) for watching TV shows, which are based on affect. Statistical analysis shows that they also display the highest impulsivity among the binge-watchers of all types. They tend to lose control, and experience severe negative affect as well as emotional reactivity. They also consider their mode of binge-watching as problematic and have reported more problematic internet use and alcohol-related problems. On the other hand, the regulated binge-watchers are motivated by emotional enhancement and characterised by low emotional reactivity and are not impulsive. Furthermore, the research conducted by Starosta, Izydorczyk, and Lizińczyk [20] also indicates that the motivation for escaping from daily life problems and coping with loneliness are most characteristic of the individuals whose binge-watching proves to be problematic. The results of this research also imply a high correlation between the motivation for escape and emotional regulation. It seems understandable that people who binge-watch excessively may use this behaviour as a form of emotional regulation. Similar conclusions have also been formulated in other studies [18,19,21,31].

### 3.7. Risks Related to Excessive Binge-Watching

The major risk behind the excessive binge-watching behaviour is the probability of developing symptoms of behavioural addiction. Multiple studies show that using binge-watching to obtain instant gratification and to regulate emotions is a maladaptive coping strategy characteristic of behavioural addictions such as problematic internet/computer use, gambling, and social media addiction [18,19,30,31]. The motivations driving problematic binge-watchers are the urge to escape from reality, dealing with loneliness, habit, or passing the time [20,31,40]. The research conducted by Merill and Rubenking [36] also indicates a statistically significant relation between binge-watching and procrastination. The results of the study imply that reward motivation and high tendency to procrastination positively predicts high frequency of binge-watching. Moreover, excessive binge-watchers display other symptoms of behavioural addiction, such as loss of self-control, urgency, regret, neglect of duties, negative social and health consequences, lying, or even symptoms of withdrawal such as anxiety, nervousness, rage, and concentration difficulties [18,19,20,30]. Numerous surveys imply that binge-watching may entail a sacrifice of some part of the viewer’s life and may become the main source of entertainment and positive affect [31]. It is also connected with the feeling of regret or guilt, especially if the binge-watching sessions are usually unplanned and uncontrolled [18,22]. Further research results highlight that binge-watchers can automatically anticipate regret and goal conflict [36,38]. The goal conflict between entertainment and obligations may diminish all positive effects of media consuming and become a source of negative emotions. However, the studies conducted by Merill and Rubenking [36] imply that regret was a negative predictor of binge-watching frequency. The research conducted by Castro, Rigby, Cabral, and Nisi [39] indicates that individuals feel more unhappy after a binge-watching session, which might be a result of their return to reality after having been engaged in a highly entertaining and immersive activity. Furthermore, individuals who display a tendency toward problematic binge-watching may also decide to sacrifice their sleep to watch another TV series episode, which can lead to fatigue, lower efficiency at work or school, and sleep deprivation [24,25,41]. Exelmans and Van den Bulck [25] emphasise the impact of binge-watching on sleep quality. Excessive binge-watchers experience sleep of poorer quality and show more symptoms of insomnia than regular TV watchers. Their results show that cognitive pre-sleep arousal is a significant mediator in the above-mentioned relationships. On the contrary, the regression analysis results obtained by Oberschmidt [24] imply that binge-watching is not a significant predictor of sleep quality and quantity. However, there is a statistically significant negative relation between binge-watching at night and sleep quantity. In consideration of these inconsistent results, there is a need for further research to determine the nature of the relation between binge-watching and sleeping patterns. As a sedentary behaviour pattern, binge-watching is also related to consumption of unhealthy food and reduced physical activity, especially among young people [32]. Furthermore, the research conducted by Ahmed [46] indicates that there is a statistically significant positive relation between the frequency of binge-watching and depression. Similar results have been obtained by Wheeler [16] who emphasises that binge-watchers are more likely to display the symptoms of depression, loneliness, and anxiety. On the contrary, Tefertiller and Maxwell [45] have not discovered any connection between self-control, depression, loneliness, and binge-watching. The relations between emotional states, emotional disorders, sleep disorders, and binge-watching undoubtedly require further research, since the results of the current research are inconsistent and reveal no clear trends in these relations. There is still too little research concerning this subject to draw explicit conclusions about the nature of such relations and the direction of the causality between binge-watching and mental health problems.

## 4. Discussion

Binge-watching has become one of the most popular ways of spending free time for people of various age groups. It is extremely popular in the population of millennials (individuals born between 1980 and 2000). Young people account for nearly 70% of the regularly binge-watching audience [10,13,26,50]. The most common definition of binge-watching is watching multiple episodes of a TV show in one sitting [1]. However, the results of the systematic review show that it is still difficult to create a consistent definition of binge-watching. The definitions addressed in this systematic review differ from one another in terms of the frequency of this behaviour, the number of episodes watched in one sitting, the episode duration, and the content.

The research on the binge-watching phenomenon focuses primarily on such conditions of binge-watching as motivation or personality traits. Some of the studies refer to the negative outcomes of excessive binge-watching and emphasise the probability risks of addiction. It may be concluded that binge-watching can be just entertainment, a way of spending free time, and relaxation [4,20,21,29,30,31,38,42,43,51,52]. According to some other research, the social and cognitive motivations are also significant for binge-watchers [20,31,34,37]. Furthermore, immersion and transportation into the narrative may be the underlying mechanisms of binge-watching, which is also typical of reading or playing video games [4,35,42,53,54]. However, excessive binge-watching can also be a serious problem including symptoms characteristic of behavioural addiction or can be related to other mental disorders, such as depression or anxiety and sleeping problems [16,18,19,20,21,30,33]. However, due to the inconsistency of the results, it is hard to determine the nature of the relationship between binge-watching and mental health problems. The similarity between excessive binge-watching and video game or internet addiction seems to be enormous in terms of psychological conditions. Neuroticism and introversion, low self-esteem, isolation, and low conscientiousness are the main predictors of behavioural addition to new media [49,55,56]. People who display a tendency for excessive binge-watching seem to be also characterised by a high level of neuroticism and a low level of conscientiousness [28]. Furthermore, excessive forms of some behaviour patterns used as a maladaptive strategy to cope with daily life problems and to regulate affect are characteristic of all forms of behavioural addiction, including binge-watching [18,19,20,21,30,51]. Moreover, impulsivity, urgency, and need of instant gratification are the main predictors of all behavioural addiction [18,28,30,57,58,59]. It is also important to mention that research implies that excessive binge-watching features all the symptoms of addiction, such as loss of control, guilt, negative social or health consequences, neglect of duties, loss of other forms of gratification, or withdrawal [18,19,20,21,30,39,60,61,62]. Multiple studies emphasise that people who play video games or use the internet excessively experience more intense symptoms of depression, anxiety, social anxiety disorder in particular, as well as sleeping disorders, and are exposed to a higher risk of substance addiction and lack of self-control [63,64,65,66,67,68,69]. Some studies indicate that excessive forms of binge-watching are related to a higher level of depression, loneliness, and anxiety [16,33,45,46]. One can assume that problematic binge-watching would also be related to the above-mentioned mental problems. However, the relation between excessive binge-watching and mental disorders is still unclear and requires further research. It can be surmised that this relation could be bidirectional in nature, just as with addiction to video games and the internet, where these kinds of behaviour are both predictors and consequences of depression or anxiety disorders [70]. Binge-watching is a highly entertaining behaviour, while its immersive character may create an opportunity for high cognitive and emotional engagement into the narrative, which could lead to the loss of the control over the time spent on this activity [3]. It can be a very positive and highly exciting experience; however, it is important to remember that the said immersive character of binge-watching can also be conducive to the risk of addiction. Furthermore, the Uses and Gratification Theory emphasises that using new technologies, including binge-watching, can be a very entertaining activity that caters to an individual’s specific needs [19,20,30,42,71].

## 5. Limitation of the Research

Most of the studies covered by the systematic research addressed in this paper were conducted in the USA (*n* = 16) or in European countries (*n* = 10), including Belgium (*n* = 3), the Netherlands (*n* = 2), Poland (*n* = 1), Portugal (*n* = 1), the United Kingdom (*n* = 1), Germany (*n* = 1), and Hungary (*n* = 1). This seems perfectly understandable since binge-watching was popularised by an American company—Netflix. However, Netflix and other streaming platforms are currently available in most countries around the world, which is why it is also important that more research on the behavioural patterns and psychological conditions of binge-watching be conducted on different populations worldwide. Secondly, women constitute the majority of those surveyed under most of the studies referred to in this paper, which is why it may be assumed that women binge-watch more than men [20,32]. However, it is important to emphasise that, compared to men, women also seem to have been more willing to participate in the surveys. In further research, it would be important to examine the male population in greater detail in order to explore the binge-watching behaviour patterns typical of men and to compare them with women. Another limitation of the research is that most participants of the studies covered by this systematic review were in their twenties. It can be explained by the fact that young adults are the main consumers of streaming platforms and that most of the surveys were conducted online. It can be assumed that young adults seem to be the most accessible research group. Furthermore, this could be related to the fact that the majority of the research participants were students. Undergraduates are by far the most accessible research group, and one may also assume that they simply have enough time to binge-watch numerous TV shows. However, it is important not to generalise about the results obtained by studying only the group of students among the entire population. With regard to the foregoing, it seems important to conduct more research involving people of different age groups as well, including adolescents, middle-aged people, and seniors, as well as people of diverse professional status. Another limitation to the research is the lack of randomised and longitudinal studies on the psychological conditions of binge-watching, which would be characterised by utmost validity and reliability. The current research on the relation between excessive binge-watching and mental disorders, such as depression, anxiety, and insomnia, presents inconsistent results. Furthermore, one must face the difficulty in establishing the direction inherent in these interrelations. The research focused on the subject of video games and internet addiction also highlights the problem of determining whether these excessive behaviour patterns are predictors or effects of mental disorder [70]. There is a need for further research on the nature of the relation between mental disorders and binge-watching. The last limitation of the studies addressed in the paper is the lack of consensus on how to define binge-watching. A proper definition of this construct would be more than welcome. Some research concerning the fast-developing psychosocial phenomena such as binge-watching as well as its psychological and social conditions, the population diversity in terms of age, nationality, and professional status could lead to better understanding of this behaviour. It would also prove helpful in identifying the differences between “normal”, i.e., highly entertaining and healthy binge-watching, and excessive and compulsive binge-watching, bearing the symptoms of behavioural addiction.

## 6. Conclusions

To summarise the foregoing elaborations, binge-watching can be both harmonious and highly entertaining as well as an obsessive and compensatory behaviour. People are driven by multiple motivations for binge-watching, including those of a social and cognitive nature, by transportation into the narrative, or willingness to pass the time, but also to escape daily life problems, to regulate negative emotions, and to gain instant gratification. The research implies that, particularly, the excessive forms of binge-watching can involve symptoms of addiction, such as lack of control, negative health and social effects, feeling of guilt, and neglect of duties. Flayelle et al. [3] have emphasised that it is important to distinguish the healthy way of consuming TV series from the problematic and excessive forms of binge-watching. In consideration of all the foregoing reasons, i.e., the risk of negative outcomes of excessive binge-watching, the increasing popularity of the phenomenon, especially among young people, and the similarities with other behavioural addictions, it seems important to continue the research on both unproblematic and problematic binge-watching, as well as on the relation between this activity and mental disorders. Further studies addressing diverse population groups would also prove helpful for better understanding of this phenomenon and could potentially lead to improved prevention and therapeutic care.

## Figures and Tables

**Table 1:** Description of the studies covered by the systematic review.

Study	Country of Research	Research Group (% of Women)	Variable Measured	Method
Conlin (2015) [4]	USA	379 (57%)	Demographics	Online survey
			Transportability	
			Fantasy Empathy	
			BW method	
			Motivation	
			Narrative Engagement	
			Character identification	
			Transportation	
Wheeler (2015) [16]	USA	186 (58%)	Motivation	Survey
			Affinity	
			Viewing habits	
			Attachment	
			Loneliness	
			Depression	
			Psychological well-being	
Riddle et al. (2018) [17]	USA	171 (75%)	Demographics	Online survey
			BW behaviour	
			Impulsivity	
Flayelle et al. (2019A) [19]	Belgium	6556 (78%)	Demographics	Online survey
			BW motivation	
			BW engagement	
			Positive and negative affect	
			Compulsive internet	
			Alcohol use	
			Nicotine dependence	
			Problematic BW symptoms	
Starosta, Izydorczyk, Liziński (2019) [20]	Poland	1004 (85%)	Motivation	Online survey
			BW behaviour	
			Emotional reactions	
			Lies	
			Loss of control and neglect of duties	
			Preoccupation	
			Negative health consequences	
			Negative social consequences	
			Demographics	
Walton-Pattison, Dombrowski, Presseau (2018) [22]	UK	86 (67%)	Self-efficacy	Online survey
			Goal conflict and facilitation	
			Anticipated regret	
			Expectation	
			BW behaviour	
Oberschmidt (2017) [24]	Netherlands	329 (72%)	General compulsory health beliefs	Online survey
			Sleep	
			BW behaviour	
Exelmans, Van den Bulck (2017) [25]	Belgium	463 (62%)	Sleep quality	Online survey
			Insomnia	
			Fatigue	
			Pre-sleep arousal	
			Binge-viewing	
Govaert (2014) [28]	Netherlands	197 (66%)	Demographics	Online survey
			BW behaviour	
			Impulsiveness	
			Personality traits	
			Coping mechanism	
Pittman, Sheehan (2015) [29]	USA	262 (62%)	Motivation	Online survey
			BW behaviour	
			Demographics	
Flayelle et al. (2019C) [30]	Belgium	4039 (80%)	Motivation	Online survey
			Impulsivity	
			Emotional reactivity	
			Sociodemographics	
			TV series watching patterns	
			BW engagement	
			Symptoms of problematic BW	
			Affect	
			Problematic internet use	
			Alcohol-related problems	
Panda, Pandey (2017) [31]	USA	229 (56%)	Demographics	Online survey
			Motivation	
			Gratification	
			Intention to spend more time BW	
Spruance et al. (2017) [32]	USA	500 (58%)	Demographics	Online survey
			BW behaviour	
			Diet	
			BMI	
			Physical activity	
Boudali, Bourgou, Jouini, Belhadj (2017) [33]	Tunis	50 (NI)	BW behaviour	Survey
			Depression	
			Anxiety	
Shim, Kim (2018) [34]	South Korea	785 (53%)	Demographics	Online survey
			Need for cognition	
			Sensation seeking	
			BW behaviour	
			Motivations for BW	
Walter, Murphy, Rosenthal (2018) [35]	USA	865 (90%)	Demographics	Online survey
			Narrative persuasion—attitude toward alcohol	
			Identification with characters	
			Method of BW	
Merill, Rubenking (2019) [36]	USA	651 (64%)	Demographics	Online survey
			BW frequency	
			BW duration	
			Self-regulation	
			Self-control	
			Enjoyment	
			Reward watching	
			Procrastination	
			Regret	
Tóth-Király et al. (2017) [37]	Hungary	Study 1—740 (72%) Study 2—944 (50%) Study 3—1520 (72%)	Demographics	Online survey
			Series watching engagement	
			Problematic BW	
			Series watching passion	
Granow, Reinecke, Ziegele (2018) [38]	Germany	499 (67%)	Demographics	Online survey
			BW behaviour	
			Goal conflict	
			Guilt	
			Autonomy	
			Recovery experience	
			Vitality	
			Entertainment experience	
Castro, Rigby, Cabral, Nisi (2019) [39]	Portugal	13 (38%)	Demographics	Online survey
			BW behaviour	
			Motivation	
			Affect states	
Sung, Kang, Lee (2018) [40]	USA	292 (76%)	Demographics	Online survey
			BW behaviour	
			Motivation	
Chamblis et al. (2017) [41]	USA	62 (69%)	Demographics	Survey
			Social distraction	
			Academic distraction	
Pittman, Steiner (2019) [42]	USA	800 (44%)	Demographics	Online survey
			BW behaviour	
			Motivations and outcome for a specific type of viewing behaviour	
			Personality	
			Multitasking	
			Regret	
Rubenking, Bracken (2018) [43]	USA	797 (56%)	Demographics	Online survey
			BW behaviour	
			Emotional regulation	
			Viewing frequency	
			Motivation	
			Self-efficacy	
			Self-control	
Conlin, Billings, Averset (2016) [44]	USA	160 (49%)	Demographic	Survey
			Viewing behaviour	
			FOMO	
Tefertiller, Maxwell (2018) [45]	USA	215 (46%)	Demographics	Survey
			BW behaviour	
			Depression	
			Anxiety	
			Loneliness	
			Self-control	
			Emotion/affect	
			Hedonic enjoyment	
Ahmed (2017) [46]	United Arab Emirates	260 (72%)	Demographics	Survey
			BW behaviour	
			Depression	
			Loneliness	
Schweidel, Moe (2016) [47]	USA	9873 (NI)	Viewing behaviour	Online survey
			Advertising exposures	
Erickson, Dal Cin, Byl (2018) [48]	USA	77 (76%)	Demographics	Experiment
			BW behaviour	
			Narrative transportation	
			Parasocial relationship	

BW—binge-watching, NI—no information, FOMO—fear of missing out, BMI—body mass index.
